# Magnetic resonance imaging/ultrasound fusion-guided transperineal prostate biopsy: Protocol for a clinic-based surgical technique

**DOI:** 10.14440/jbm.2025.0115

**Published:** 2025-03-07

**Authors:** Ilias Giannakodimos, Napoleon Moulavasilis, Aris Kaltsas, Dionysios Mitropoulos, Michael Chrisofos, Konstantinos Stravodimos, Evangelos Fragkiadis

**Affiliations:** 1 Third Department of Urology, Attikon University Hospital, School of Medicine, National and Kapodistrian University of Athens, Athens 12462, Greece; 2 Department of Urology, Laikon General Hospital, National and Kapodistrian University of Athens, Athens 11527, Greece

**Keywords:** Prostate biopsy, Transperineal approach, Fusion magnetic resonance imaging/ultrasound, Prostate cancer, Diagnostic protocol, Infection reduction

## Abstract

**Background:**

Prostate biopsy is a crucial diagnostic tool for detecting clinically significant prostate cancer (csPCa). Traditional transrectal ultrasound (TRUS)-guided biopsy methods are often associated with an increased infection risk of infection and limited accuracy, particularly when diagnosing anterior lesions of the prostate gland.

**Objective:**

This article presented a structured protocol for performing transperineal fusion magnetic resonance imaging/ultrasound (MRI/US) prostate biopsy, highlighting its advantages over the TRUS approach. Our study included biopsy-naïve patients with elevated prostate-specific antigen levels or abnormal digital rectal examination findings, all of whom underwent pre-biopsy multiparametric MRI to guide targeted biopsies. The key objectives of this protocol were to improve the detection rates of csPCa, minimize infection risk, and standardize a transperineal technique that combines both systematic and targeted biopsies. In addition, we provided details on patient preparation, equipment requirements, procedural steps, and follow-up protocols to ensure the safety and effectiveness of the procedure. This protocol aims to serve as a guideline for institutions to adopt MRI/US fusion-guided transperineal biopsy, thereby enhancing diagnostic accuracy and patient safety.

**Conclusion:**

The transperineal fusion MRI/US biopsy protocol enhances diagnostic accuracy, particularly for anterior lesions, while reducing infections risks. Combining targeted and systematic biopsies improves detection rates of csPCa and offers a standardized, safe approach for clinical implementation.

## 1. Introduction

Men with elevated prostate-specific antigen (PSA) levels or suspicious findings from digital rectal examination (DRE) typically undergo traditional transrectal ultrasound (TRUS)-guided prostate biopsy, which involves the collection of at least 12 cores, with six taken from each prostate lobe.^1^ However, TRUS-guided biopsy presents several limitations, including an increased risk of post-biopsy infection and reduced detection rates for certain types of lesions, particularly in the anterior prostate.^2^ In contrast, the transperineal approach has demonstrated superior diagnostic accuracy for anterior lesions and significantly reduces the risk of infection compared to the TRUS approach.^2^,^3^ In a study conducted by Pepe and Pennisi^4^ involving 8,500 men who underwent transperineal prostate biopsy, prostate cancer (PCa) was found in 37.1% of cases. Clinical complications occurred in 35.9% of the patients; notably, only 1.5% required hospital admission. As a result, the European Association of Urology (EAU) PCa Guideline Panel now strongly recommends the transperineal method as the preferred approach for prostate biopsy.^1^

With advancements in imaging technology, multiparametric magnetic resonance imaging (mp-MRI) has become an essential tool for identifying suspicious prostate lesions. It enables targeted biopsies, thereby reducing the need for unnecessary biopsies when no suspicious areas are detected.^5^ Recent studies comparing MRI-targeted biopsies to traditional systematic biopsies have found that targeted biopsies alone can achieve similar or even higher rates of clinically significant cancer detection.^6^,^7^ Current guidelines recommend a combined approach, advocating for the acquisition of 12 systematic biopsy cores along with 3 – 5 targeted biopsy cores for suspected lesions, with the number of targeted cores depending on lesion size.^1^ MRI-targeted biopsy can be performed using various methods, including cognitive guidance, MRI/ultrasound (US) fusion software, or direct in-bore guidance.^1^ Interestingly, no significant difference in the detection rate of PCa (International Society of Urological Pathology [ISUP] grade >2) has been reported between these techniques, and thus, the choice of method should be based on the urologist’s experience and the resources available at each institution.^8^ Another benefit of advancing imaging technology is the integration of prostate-specific membrane antigen (PSMA) positron emission tomography (PET) with computed tomography (CT) in everyday clinical practice. A systematic review of 12 studies evaluating the role of ^68G^a-PSMA PET/CT for initial staging found increased sensitivity (range 33 – 99%) and specificity (per-lesion 82 – 100%, and per-patient 67 – 99%), demonstrating higher detection rates compared to bone scans or CT.^9^ However, the prognostic role of this new imaging modality remains unclear, and the optimal management of patients with metastases detected only through PSMA PET/CT has yet to be determined.^1^

MRI-targeted transperineal biopsies improve the detection of clinically significant PCa (csPCa), as defined by any Gleason pattern 4 disease, by approximately 1.28 times compared to transrectal approaches. Transperineal methods also detect 2.46 times more anterior lesions.^10^ In addition, a meta-analysis revealed that MRI-targeted transperineal biopsies resulted in an infection rate of only 0.6% without routine antibiotic prophylaxis, demonstrating the technique’s reduced infection risk.^11^

Despite the clear clinical advantages of the transperineal approach, its adoption in routine clinical practice remains limited. Many urological departments lack the specialized equipment and technical expertise needed to implement MRI/US fusion-guided transperineal biopsies. Furthermore, only a few studies offered comprehensive, standardized protocols that detail the necessary technical equipment, procedural steps, and practical guidance to minimize technical errors during transperineal biopsy.^12^

This article aimed to address these gaps by presenting a detailed, well-structured protocol for managing and following up patients undergoing transperineal fusion MRI/US biopsies. We outlined the necessary equipment and essential surgical skills, providing a practical guide for urologists to optimize diagnostic outcomes while ensuring procedural safety and consistency in clinical practice.

## 2. Design and methods

### 2.1. Study population

This ongoing study is a prospective cohort study that includes biopsy-naïve patients with elevated PSA levels who undergo transperineal fusion MRI/US biopsy. The aim of this study was to provide a well-structured protocol for performing transperineal MRI/US fusion-targeted and systematic prostate biopsy, as proposed by our clinic, and to describe the technical equipment and surgical skills required for this procedure.

This study intended to recruit biopsy-naïve men referred to the Urology Department with suspected PCa, based on elevated age-specific PSA levels or abnormal DRE findings, who were suitable for pre-biopsy MRI and prostate biopsy. The specific inclusion criteria were as follows: men with clinical suspicion of PCa based on a positive DRE, regardless of PSA findings; men aged >18 years with abnormally elevated PSA levels suggestive of PCa, as defined in our institution (PSA >4 ng/mL); men with suspicious findings on pre-biopsy mp-MRI (prostate imaging reporting and data system [PI-RADS] score 3 – 5) using an MRI scanner with a field strength of ≥1.5 Tesla; and participants must be capable of undergoing all the procedures described in the protocol, providing informed consent, and understanding written English. Exclusion criteria include a history of previous prostate biopsy, findings suggestive of extensive local disease (either by physical examination, DRE, or elevated PSA levels), a positive pre-biopsy urine culture, symptoms suggestive of concurrent or recent urinary infection, a history of immunocompromise, or an inability to be placed in the lithotomy position. The detailed inclusion and exclusion criteria for the study are summarized in Table 1.

**Table 1:** Inclusion and exclusion criteria of the study

Study inclusion criteria	Study exclusion criteria
• Men with clinical suspicion of prostate cancer based on a positive digital rectal examination	• Men with PSA levels within the normal range for their age
• Men with suspected prostate cancer and abnormal PSA levels for their age	• Men receiving treatment for prostate cancer
• Men with suspicious findings on multiparametric MRI	• Men with clinical suspicion of prostate cancer but without evaluation through multiparametric MRI
• Men capable of undergoing any procedure included in the study protocol	• Men with contraindications for prostate biopsy
• Men who consented to participate in the study	• Men unable to undergo any procedure included in the study protocol
• Men aged >18 years	• Men who did not consent to participate in the study

Abbreviations: MRI: Magnetic resonance imaging; PSA: Prostate-specific antigen.

### 2.2. Primary/secondary endpoints

The primary outcome was the presence of the PCa and csPCa identified through transperineal prostate biopsy. The secondary outcomes include the incidence of infection and post-biopsy complications, histological parameters after biopsy, and patient-reported outcomes, as measured by the International Prostate Symptom Score (IPSS) questionnaire.

### 2.3. Trial overview and follow-up

This study involved a patient visit to the Urology Department. Each participant arrived at the clinic in the morning, one day before the scheduled biopsy, after fasting for 12 – 14 h. During the visit, demographic data and a complete medical history, including comorbidities and medications, were recorded. Body measurements (weight, height) were taken, and body mass index (BMI) was calculated. Blood samples were collected for general tests (complete blood count, biochemistry, coagulation factors, PSA, glycosylated hemoglobin, etc.) and for storing biological materials for future use. A urine test and culture were also performed. A full clinical examination, including DRE, was conducted to locate any suspicious lesions in the prostate. Finally, the IPSS questionnaire was completed with the guidance of a specialized urologist to evaluate the patient’s lower urinary tract symptoms before the biopsy. After the initial visit, participants remained in the hospital until the scheduled biopsy was performed.

On the 7^th^-day post-biopsy, an urologist from our department contacted participants by phone to inquire about any discomfort experienced after the biopsy. Specifically, patients were asked if they suffered from hematuria, rectal bleeding, hemospermia, urinary retention, fever, required additional antibiotic treatment, or encountered any complications that necessitated hospital care. Patients were also asked to complete the IPSS questionnaire again, which they had previously filled out during their hospital stay so that their post-biopsy scores could be compared with those recorded before the procedure.

One month after the biopsy, a second phone call was made to gather information on whether the previously mentioned symptoms persisted. Participants were also advised to contact the Pathology Department for the results of the histopathological analysis of their biopsy. According to the study protocol, no additional visits were scheduled after the biopsy. Men who underwent transperineal biopsy would be informed of their biopsy results by an urologist at our clinic and will receive appropriate management based on the pathological findings reported.

### 2.4. Pre-biopsy multiparametric-MRI

**Figure 1:**
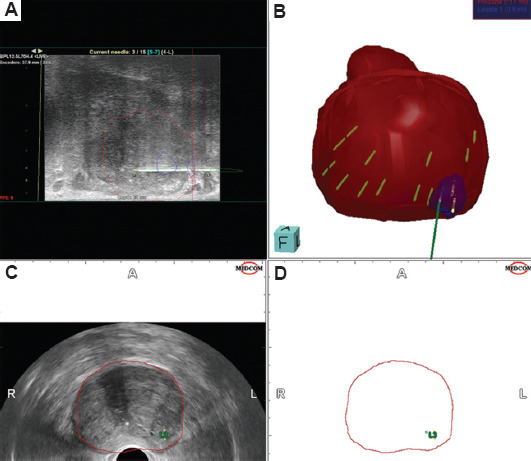
The process of magnetic resonance imaging/ultrasound (MRI/US) fusion-guided biopsy, combining targeted and systematic biopsies. (A) Targeted prostate biopsy of the suspected lesion obtained by needle under MRI/US fusion. (B) Three-dimensional model of the prostate (red), the suspected lesion (blue), and both systematic and targeted biopsies obtained. (C and D) Planning of the prostate borders and identification of the location of the suspected lesion. Red-marked area: prostate; blue-marked area: lesion; A: Anterior, L: Left; P: Posterior; R: Right.

All recruited male patients had to undergo mp-MRI before biopsy to detect suspicious lesions. The mp-MRI should be performed on a 1.5-Tesla or higher MRI scanner, with a radiological report provided by a qualified radiologist, according to PI-RADS. The location of radiologically suspicious lesions would guide targeted fusion MRI/US transperineal biopsy. An example of a fusion MRI/US transperineal systematic and targeted biopsy performed in our CLINIC is presented in Figure 1.

### 2.5. Technique of MRI/US fusion transperineal biopsy

#### 2.5.1. Technical equipment and set-up

To perform the transperineal biopsy, basic equipment is required, including an operating table with lithotomy stirrups, steppers, brachytherapy grid, a US machine with a transrectal US probe, core biopsy needles, biopsy cups with formalin, and separate cups for each core obtained. A list of the required technical equipment is presented in Table 2.

**Table 2:** Technical equipment and patient preparation

Technical equipment and set-up
• US machine
• Transrectal US probe
• Core biopsy needles
• Biopsy cups with formalin
• Operating table
• Lithotomy stirrups
• Steppers
• Brachytherapy grid
Preparation of the patient
• Scrotum elevation
• Scrotum secured with tapes
• Perineum cleansing with 7.5% povidone-iodine solution
• General anesthesia
• Prophylactic antibiotics administration
• Lithotomy position

Abbreviation: US: Ultrasound.

#### 2.5.2. Preparation of the patient

All patients were subjected to general anesthesia. Prophylactic antibiotics, typically a 1 g bolus of amikacin, were administered intraoperatively. The patient was positioned in the lithotomy position on the operating table, with the anus aligned with the edge of the table. The table height was adjusted so that the patient’s anus was at the level of the surgeon’s elbow. The scrotum was elevated and secured with tape to provide clear access to the perineal area, which was then shaved of excess hair. The perineum was prepared using a 7.5% povidone-iodine solution (Betadine). A stepper device was placed at the end of the operating table to facilitate the attachment of a sampling brachytherapy grid at the perineal level and the US probe at the rectal level. The patient preparation steps are summarized in Table 2.

#### 2.5.3. Transperineal technique

A US probe, surrounded by xylocaine gel, was inserted into the rectum. The gland was visualized fully, including both axial and sagittal fields, to identify surgical landmarks (such as the urethra) and estimate prostate volume. The mp-MRI/US fusion software integrates preoperative mp-MRI results with real-time TRUS imaging, allowing for the mapping of the prostate lobes. This mapping helps to identify prostate landmarks and borders. Furthermore, preoperative prostate mapping aims to locate prostatic suspicious lesions identified in the mp-MRI (PI-RADS >3) and target prostate regions for subsequent biopsies, as previously described. In our technique, another important landmark for prostate mapping was the apical prostatic urethra, located near the apex of the prostate, which helps guide the collection of bilateral biopsies near the urethra. This area is crucial for anastomoses during radical prostatectomy, and the presence of cancer in this region may influence the urologist’s surgical decisions. Biopsy cores were obtained through the holes of the brachytherapy grid to ensure targeted sampling of the designated areas. The needle was advanced under direct visualization, and complete small 3 – 10° probe rolls (micro rolls) were performed to guide the needle tip into the intended target. After obtaining all targeted and systematic biopsies, the needle and US probe were removed. Pressure was applied on the perineum with a towel for 1 – 5 min until hemostasis was achieved at the puncture site.

### 2.6. Histological report

The transperineal fusion MRI/US biopsy involved a total of 12 systematic biopsy cores from both prostatic lobes and 2 – 3 targeted cores from each suspicious lesion, depending on lesion size. For the systematic biopsies, cores were taken from six sectors, with two biopsy cores from the anterior, middle, and posterior sectors of the prostate gland, on both the left and right sides. Specifically, the biopsy cores taken from the apex of the prostate were collected near the prostatic urethra to further evaluate any lesions in this critical area. This region is crucial for the anastomosis during radical prostatectomy, and the presence of cancer here may affect the urologist’s surgical decision. In addition, 2 – 5 (average three) targeted biopsy cores were harvested from each significant target lesion observed on the pre-biopsy MRI, depending on the size of each lesion. All biopsy cores were sent to the Pathology Department in separate containers for evaluation of the pathological findings of each distinct biopsy core.

### 2.7. Statistical analysis

All data were entered into an electronic database for statistical processing. The statistical analyses were conducted using the STATA 15.0 software package, with a significance level set at a *p* < 0.05.

The normality of continuous variables was assessed using the Kolmogorov-Smirnov test. Parametric tests were applied to variables with a normal distribution, while non-parametric tests were employed for variables without a normal distribution. Descriptive statistics report normally distributed quantitative variables as means ± standard deviations, whereas non-normally distributed variables were presented as medians with interquartile ranges (25 – 75%). For comparing normally distributed quantitative variables between study groups, the paired *t*-test was used, and for non-normally distributed variables, the Mann-Whitney U test was employed. The comparison of qualitative variables between the study groups was made using the Chi-square (χ²) test.

Finally, univariate and multivariate logistic regression analyses were conducted, with dependent variables being the occurrence of cancer or various complications. Independent variables included targeted versus non-targeted biopsy, the number of biopsies, biopsy-related characteristics, and somatometric and clinical features of the patients.

## 3. Results

Recruitment for this study began on January 12, 2024, and the main trial is currently in progress. To date, 110 participants have met the inclusion criteria and undergone transperineal MRI/US fusion biopsy. Feedback regarding post-biopsy complications and follow-up communication for further management has been obtained from 96.4% (106 patients) of the included participants. Among the four lost patients, three did not respond to telephone communication, while one refused to participate in the follow-up and was excluded from the study.

Out of the 110 participants who satisfied the inclusion criteria, complete data on post-biopsy follow-up and histological findings were available for only 52 patients. The mean age of patients was 67.7 ± 6 years, with a mean PSA level of 7.8 ng/mL, a mean prostate volume of 67 mL as measured on mp-MRI, and a mean BMI of 27 kg/m^2^. Regarding demographic data, 28.6% of participants had diabetes (14 patients), 26.5% were receiving anticoagulant therapy (13 patients), 19.5% had a positive DRE (19.5%), and 10.2% (5 patients) had a family history of PCa. In total, 69.4% (34 patients) had one suspected lesion, 26.5% (13 patients) had two suspected lesions, and only two patients had more than two lesions detected on mp-MRI.

With regard to the incidence of post-biopsy complications, 64.6% (31 patients) developed hematuria, 62.5% (25 patients) had hemospermia, and 6.25% (3 patients) experienced bloody stools. None of the patients reported urinary retention, required further hospitalization, or died.

Concerning diagnostic accuracy, 32 patients were diagnosed with PCa, 66.7% (32 patients) of whom were diagnosed after targeted biopsy, and 39.6% (19 patients) after systematic biopsy. csPCa was identified in 52.2% (26 patients) of the participants, 47.9% (23 patients) following targeted biopsy, and 27.1% (13 patients) after systematic biopsy.

## 4. Discussion

As aforementioned, the EAU guidelines for PCa strongly recommend performing prostate biopsies through the transperineal route to reduce the incidence of post-biopsy infections.^1^ Recent studies in the literature indicated a shift from TRUS to transperineal biopsy in clinical practice, aiming to lower the infection risk associated with TRUS biopsy.^13^ However, the majority of institutions still lack both the necessary technical equipment and surgical experience to allow for this change. In this study, we presented a well-structured protocol for performing transperineal fusion MRI/US biopsy of the prostate, detailing the equipment and surgical technique used in our department. To our knowledge, we are the first to propose that two of the 12 systematic biopsies be taken from the apical prostatic urethra, bilaterally, to provide additional information for the surgeon regarding the area of potential vesicourethral anastomosis. Furthermore, our protocol includes a comprehensive follow-up program, which has resulted in a high acceptance rate for re-evaluation. To date, 96% of the included patients have responded to follow-up communication, while only three patients lost to the follow-up and one declined post-biopsy communication.

Regarding post-biopsy infection complications, a systematic review by Bennet *et al.*,^14^ including 165 studies and 162,577 patients, reported a sepsis rate of 0.1% for transperineal biopsies and a rate of 0.9% for transrectal biopsies. Interestingly, a meta-analysis of 4,280 men randomized between transperineal and TRUS biopsies found no significant differences in complication rates, although the number of patients with septic incidents was small.^15^ While the role of the transperineal route in reducing post-biopsy sepsis has been documented, uncertainty lingers regarding whether it provides superior cancer detection rates compared to the transrectal approach. Interestingly, in a systematic review and meta-analysis comparing MRI-targeted transrectal biopsy to MRI-targeted transperineal biopsy, the transperineal approach demonstrated an increased detection rate (86%) compared to the transrectal approach (73%), especially for anterior prostatic lesions.^2^ On the contrary, a meta-analysis by Xiang *et al*.^16^ showed that the transperineal approach achieved a diagnostic accuracy comparable to that of the transrectal route. Although the EAU guidelines recommend the transperineal approach as the optimal technique principally due to its lower post-biopsy infectious rates, it remains debatable whether MRI-targeted transperineal biopsy offers a diagnostic advantage over the transrectal route in detecting csPCa.^1^ Of note, in a randomized control trial conducted by Hu *et al.*,^17^ the detection rate of clinically significant cancer was similar between the two routes (53% for transperineal vs. 50% for transrectal). A systematic review and meta-analysis by Tu *et al*.^2^ found that in patients with suspicious lesions in the mp-MRI, targeted biopsies through the transperineal route yielded a higher detection rate (62.2%) compared to the transrectal route (41.3%). In the same study, when systematic and targeted biopsies were combined, the transperineal approach was associated with an increased incidence (91.3%) of csPCa compared to the transrectal approach (72.2%).^2^ In contrast, a meta-analysis by Uleri *et al*.^18^ found no statistically significant difference in MRI-targeted biopsy outcomes between the transrectal and transperineal approaches. Interestingly, in the same study, MRI-targeted biopsy through the transperineal route was associated with higher detection rates of csPCa anterior lesions (odds ratio = 2.17, *p* < 0.001) and apical lesions (odds ratio = 1.86, *p* = 0.01), while no statistically significant difference was found for posterior lesions.^18^

Regarding the role of pre-biopsy MRI and targeted biopsy, the EAU guidelines strongly recommend preoperative MRI in men with organ-confined disease to identify suspicious prostate lesions.^1^ According to the PRECISE trial, a prospective study that compared the detection rates of systematic biopsy to MRI-targeted biopsy, targeted biopsy was found to be non-inferior for detecting ISUP grade group ≥2 cancers.^7^ A meta-analysis that included biopsy-naïve patients with suspicious lesions on MRI revealed that MRI-targeted biopsy detected significantly more ISUP grade group ≥2 tumors compared to systematic biopsy.^1^ As a result, MRI-targeted biopsy appears to attain better detection rates for ISUP grade ≥ tumors compared to systematic biopsy.^1^ In addition, targeted MRI reduces the detection of non-clinically significant cancers labeled as ISUP grade 1. Specifically, the 4M trial showed that patients who underwent MRI-targeted biopsy had lower rates of ISUP grade 1 tumors compared to those who received systematic biopsy.^19^ Similar findings regarding the identification of ISUP grade 1 cancers were observed in the Precise trial.^7^

Although the value of targeted biopsy in identifying clinically significant cancer cases is well-established, it should be combined with systematic biopsies. Interestingly, integrating MRI-targeted biopsy with systematic biopsy increases the detection rates of ISUP grade ≥2 and grade ≥3 tumors by approximately 20% and 30%, respectively.^1^ On the other hand, omitting systematic biopsies in biopsy-naïve men results in the missed detection of approximately 16% and 18% of all ISUP grade ≥2 and grade ≥3 tumors, respectively.^20^,^21^ The combination of systematic biopsies and targeted biopsies remains the standard of care and is described in our technique. In our protocol, 12 systematic biopsies (six from each lobe), along with 2 – 3 targeted biopsies based on prostate size, are considered the optimal approach. Evidence from multicenter studies highlights that the MRI/US fusion-guided transperineal approach not only improves diagnostic yield but also maintains a high negative predictive value, which enhances the reliability of excluding clinically insignificant cancers, thereby reducing the risk of overtreatment.^22^ Studies have also shown that the detection of csPCa in transperineal biopsies is superior, particularly in high-risk patients with previous negative biopsies.^23^ Interestingly, we also suggest obtaining biopsies from the apical section of the prostatic urethra, an area where the vesicourethral anastomosis will occur, which will impact future surgical decisions. However, according to the EAU guidelines for PCa, urologists should consider obtaining additional perilesional biopsies while avoiding systematic biopsies in regions of the prostate without suspected lesions^1^. Specifically, Brisbane *et al*.^24^ demonstrated that 90% of systematic biopsies with csPCa findings were taken within a 10-mm radius of the MRI suspected lesion, while the detection rate of csPCa dropped as the distance from the suspected lesion increased.^24^,^25^ Nonetheless, a meta-analysis found no significant differences in the detection rates of csPCa between MRI-targeted biopsy combined with perilesional biopsy and the combination of MRI-targeted and systematic biopsies.^26^

## 5. Conclusion

A combination of targeted and systematic biopsy through the transperineal route should be the standard technique for prostate biopsies, as it improves detection rates and reduces the post-biopsy infection risk. Our study provides a well-structured protocol for performing transperineal fusion MRI/US biopsies, along with a follow-up strategy for these patients. Further well-designed protocols are warranted to detail the surgical techniques and equipment required to standardize this approach for everyday clinical practice.

## Data Availability

Data are available at 10.5281/zenodo.14691097.
